# A methodology for optimizing treatment head angle arrangement for multi-angle FLASH intensity modulated radiation therapy platforms

**DOI:** 10.3389/fonc.2025.1628281

**Published:** 2025-09-17

**Authors:** Weijie Cui, Chenlei Guo, Zhihui Hu, Yunxiang Wang, Kuo Men, Jianrong Dai

**Affiliations:** Department of Radiation Oncology, National Cancer Center/National Clinical Research Center for Cancer/Cancer Hospital, Chinese Academy of Medical Sciences and Peking Union Medical College, Beijing, China

**Keywords:** flash, treatment head angle, optimization, multi-angle FLASH therapy platform, adaptive simulated annealing

## Abstract

**Purpose:**

Flash therapy technology has been introduced, and several systems have been developed for its implementation. One such FLASH radiotherapy platform employs multiple treatment heads that deliver radiation to a target simultaneously. However, the optimal number of treatment heads and their precise angular configuration needed to best meet clinical requirements remain to be determined.

**Methods and materials:**

In this study, each treatment head angle is treated as an independent variable, and the total angular discrepancy between a set of beam directions from clinically used plans and those generated by a virtual FLASH radiotherapy platform is defined as the objective function. This problem is solved using an optimization technique known as Adaptive Simulated Annealing (ASA). The performance of the proposed optimization model was evaluated using a dataset of 69,928 beams from 8,866 intensity-modulated radiation therapy (IMRT) plans collected over a two-year period in our department. These plans represent various types of common tumors, including nasopharyngeal, breast, esophageal, lung, and rectal cancers. The total angular discrepancy was compared between the beam directions obtained through the optimized treatment head arrangement and the directions used in clinical practice.

**Results:**

For a virtual FLASH therapy platform equipped with five treatment heads, we obtained the optimized treatment head angle arrangements both with and without the constraint of an imaging system. Under the imaging system constraint, the optimized angles were 0°, 40.4°, 169.4°, 201.2°, and 239.8°, resulting in an average discrepancy of 38.9°compared to the beam directions used in the reference treatment plan cohort. Without the imaging system constraint, the optimized angles were 0°, 155.4°, 234.4°, 266.2°, and 304.8°, yielding an average discrepancy of 37.8°. In contrast, equally spaced treatment head angles produced an average discrepancy of 78.4°.

**Conclusion:**

A methodology for optimizing the treatment head angle arrangement for multi-angle FLASH radiotherapy platforms is proposed. The optimized configuration provides an effective solution for clinical applications, balancing performance with practical feasibility.

## 1 Introduction

In recent years, FLASH radiotherapy has been shown to reduce radiation-induced toxicity to normal tissues, while maintaining antitumor efficacy, when compared to conventional dose rate (CONV) radiotherapy using the same total dose (1–4). This phenomenon, known as the *FLASH effect*, has been observed across various experimental models, including those with animals (mice, zebrafish, pigs, cats) and organs (lung, gut, brain, skin), providing a promising basis for translating FLASH radiotherapy to human patients (5–8).

While the underlying mechanism behind the normal tissue sparing observed in FLASH radiotherapy continue to be explored, significant technological advancements have been made. The feasibility of inducing FLASH effects *in vivo* has been demonstrated using proton, electron, heavy ion, and x-ray systems. The thresh hold dose rate to trigger FLASH effect is widely accepted to be 40Gy/s (9). Due to ultra-high dose rate requirement of FLASH therapy, it is essential to reduce the total delivery time, ideally reducing it to sub-second levels. This duration is much shorter than typical delivery time of several minutes for modern radiotherapy techniques such as Intensity Modulated Radiotherapy (IMRT) and Volumetric Modulated Arc Therapy (VMAT).

The literature reports three primary approaches to reducing therapy time. The first approach is to deliver a single beam per fraction to eliminate intra-fraction gantry motion (10). Although different beam directions can be used across sessions, this approach clearly lacks dose conformity. The second approach involves fast mechanical motion. Ke Sheng et al. (11) proposed the Decoupled ring-collimator for ultrafast dose delivery (ROAD), which combines a fast-rotating slip-ring linac and a decoupled collimator-ring with 75 pre-shaped multi-leaf collimator (MLC) modules. In the design, the ring-source rotates clockwise at 1 rotation per second (rps), while the ring-collimator is either static or rotates counterclockwise. However, this method faces significant mechanical challenges due to the rapid rotation and entails high costs associated with the large number of MLC modules. The third approach is multi-angle FLASH delivery without mechanical motion.

To date, two approaches for achieving multi-angle FLASH therapy without mechanical motion have been reported. The first is the PHASER (12) (Pluridirectional High-energy Agile Scanning Electronic Radiotherapy) system, designed for 16-beam IMRT FLASH delivery. In this system, all treatment heads deliver beams nearly simultaneously without mechanical gantry rotation. PHASER utilizes a Radiofrequency Phased-Array Power Distribution (RAPiD) network that combines RF power from 16 klystrons to supply 16 accelerators positioned at different angles. The outputs of multiple klystrons are combined through appropriate modulation of the phases to direct the summed power to any of the 16 output ports, with a switching time of 300 nanoseconds. However, this approach necessitates multiple RF power sources and precise phase control, increasing both cost and system complexity.

The second approach is the MAX-FLASH (Multi-Angle X-ray FLASH radiotherapy) system, which draws inspiration from multiplexing techniques in modern communication systems. MAX-FLASH incorporates a compact multiplexer specifically developed for linac systems (13, 14). It employs a few RF sources to supply multiple linacs placed at different angular positions. The RF transmission path can be rapidly selected by adjusting the power source frequency, enabling simultaneous multi-angle FLASH delivery. Although MAX-FLASH typically operates with a maximum of five treatment heads—fewer than PHASER—it offers a more cost-effective and compact solution for FLASH radiotherapy, making it suitable for most hospital radiotherapy treatment rooms.

The MAX-FLASH configuration consists of five linear accelerators installed at distinct coplanar angles on a vertically oriented O-ring gantry. The gantry rotates to a predefined angle prior to irradiation and remains stationary during simultaneous beam delivery. Since the angular distribution of the treatment heads relative to the gantry is fixed, this distribution, combine with the gantry rotation angle, determines the beam directions—an essential factor influencing treatment outcomes. This differs from conventional IMRT with C-arm linacs, in which beams are delivered sequentially and arbitrary beam directions can be used. Then it raises a critical question: how can the treatment heads be optimally positioned to accommodate the diverse beam direction requirements of various tumor sites?

In conventional IMRT, beam direction selection is a critical component of the treatment planning process. Numerous strategies have been proposed to optimize beam configurations (15–17). Nevertheless, beam direction selection is typically performed by treatment planners based on their experience, and the chosen directions often vary depending on tumor location. For instance, evenly spaced beams are commonly used for centrally located tumors—such as those in the rectum, prostate, or nasopharynx—while non-uniform beam arrangements may be more suitable for tumors in other anatomical sites.

In this study, we focus on optimizing the angular arrangement of treatment heads in FLASH radiotherapy systems with a limited number of heads (e.g., MAX-FLASH). Historical beam directions from conventional IMRT treatments were used to guide the optimization process. An optimization model was developed to minimize the discrepancy between beam directions generated by the FLASH platform and those employed in clinical IMRT plans. This problem was solved using a global optimization algorithm, Adaptive Simulated Annealing (ASA), and the optimized results were compared with those obtained from equidistant treatment head configurations. The goal of this work is to provide an optimized head arrangement that better satisfies clinical beam direction requirements and enhances the applicability of FLASH radiotherapy platforms.

## 2 Methods and materials

### 2.1 Schematic design of multi-angle FLASH radiotherapy platform

**Figure 1:**
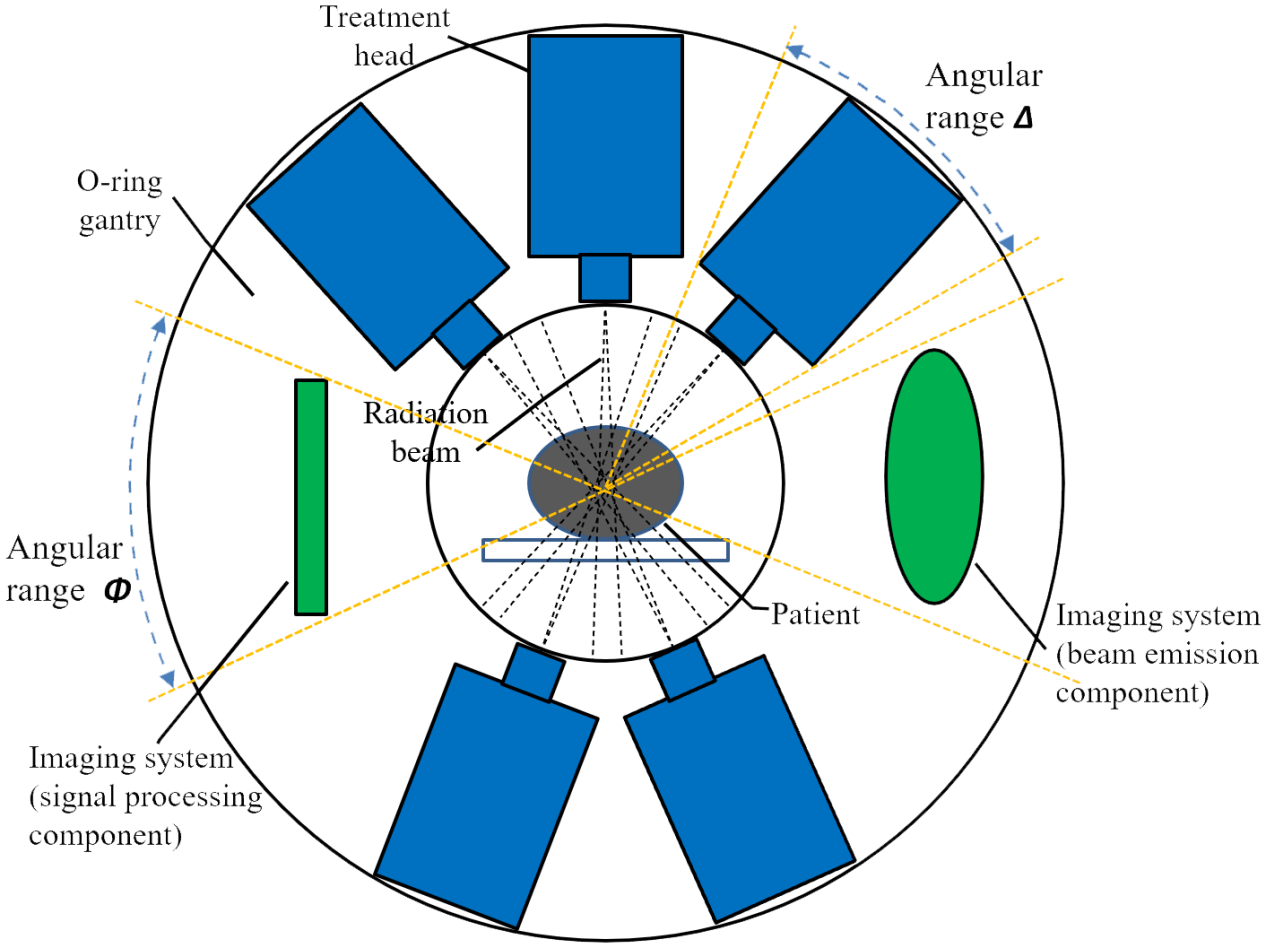
A schematic design of multi-angle FLASH radiotherapy platform. Five treatment heads and an imaging system are configured in the O-ring gantry.

Flash radiotherapy platforms with a limited number of treatment heads are still under development, and specific design details remain unclear. A schematic representation of the gantry for such a system is shown in **Figure 1**. This conceptual design features five linacs mounted vertically at different coplanar angles within an O-ring gantry. A novel distribution network is employed to rapidly switch RF power to a single terminal linac, enabling simultaneous irradiation from all treatment heads. The system is designed with a source-axis-distance (SAD) of 80 cm to allow sufficient space for the collimator and patient aperture. The gantry rotates to the desired angle prior to radiation, and remains stationary during delivery with multiple linacs. Since the angular distribution of the treatment heads relative to the gantry is fixed, the combination of this distribution and the gantry rotation angle determines the beam directions. In this study, the angular arrangement of the treatment heads is treated as an optimizing variable and is determined using the method described below. Although the schematic illustrates a five-linac configuration, the system can accommodate alternative configurations with three to six treatment heads. It should also be noted that the treatment heads are distributed non-equidistantly—that is, the angles between adjacent heads are not necessarily uniform.

**Figure 1** also illustrates two constraints for angular distribution of treatment heads. First, a minimum spacing between adjacent heads is required since each head occupies a defined angular segment. Second, an imaging system—comprising both beam emission (radiation) and signal processing components—is integrated into the O-ring gantry (18). Image-guidance is an essential component of modern radiotherapy and is expected to contribute to the precise delivery of FLASH therapy. In this study the incorporation of imaging system serves as an example of how additional constraints may be imposed on the angular arrangement of treatment head. To simulate the vertical alignment typically found between the cone-beam CT (CBCT) system and the treatment head in conventional linacs, the imaging system is positioned at a 90-degree angle relative to one of the treatment heads.

### 2.2 Rationale for optimizing treatment head angle arrangement

In the design illustrated in **Figure 1**, the FLASH treatment procedure involves selecting 1~5 heads form the five available treatment heads to irradiate the target simultaneously. When the gantry remains stationary, the five beam directions correspond directly to the fixed angular positions of the treatment heads, as shown in **Figure 1**. Once the gantry rotates, the treatment heads rotate synchronously in a coplanar fashion, producing a new set of five beam directions. If the required number of beams exceeds the number of available heads, multiple deliveries, rather than a single delivery, must be performed.

Therefore, the treatment head arrangement directly influences the achievable beam directions of a FLASH platform, making it a key consideration for system designers. Optimizing the arrangement is necessary to ensure that the system can adequately meet clinical beam requirements. However, determining the optimal configuration is challenging due to the limited understanding of the specific beam direction needs in FLASH therapy. To address this, we used historical beam directions from conventional IMRT treatments as guidance for head arrangement optimization. The total angular discrepancy across a representative cohort of clinical IMRT plans was employed as the optimization objective. This metric quantifies how well a given treatment head arrangement can reproduce clinically relevant beam directions.

### 2.3 Mathematical modeling of the angle arrangement problem

#### 2.3.1 Variables and constraints

For generality, we assume that the FLASH therapy system is equipped with *N* treatment heads capable of simultaneous irradiation. The angles corresponding to these treatment heads are denoted as *A_1_* to *A_N_* (where $0\leq A_{i}\leq 2\pi$ , 0≤i≤N ). When the O-ring gantry rotates with these heads, the specific values of *A_1_* to *A_N_* will change, but the relative spacing between the treatment heads remains fixed. Specifically, the angle relationship between treatment head *A_i_* and treatment head *A_i+1_* is given by Equation 1:


(1) $$A_{i+1}=A_{i}+\alpha_{i}\;1\leq i\leq N-1$$

where *α_i_* represents the angle spacing between midlines of adjacent heads. The angle between the first and last treatment head, *A_1_* and *A_N_*, is denoted as *α_N_*. The variables *α_1_*, *α_2_*, … *α_N_* are the optimization variables, and their values will be determined through the optimization process.

The first constraint for variables is that the sum of the angle spacings must equal 2π (the total angle around the O-ring gantry), as specified in Equation 2:


(2) $$\sum_{i=1}^{N}\alpha_{i}=2\pi$$

Next, we account for the physical size of the treatment heads. Each head occupies a certain angular range, which we denote as *Δ* degrees. Therefore, the spacing between neighboring treatment heads must be at least *Δ*, as specified in Equation 3.


(3) $$\alpha_{i}\geq \Delta ,\;for\;1\leq i\leq N$$

Additional constraints can be added to reflect the need to install the imaging system on the O-ring gantry. Specifically, we assume two imaging system constraints must be satisfied:1) The beam emission part and the signal processing part of the imaging system should occupy two angles separated by 180° and must be at least *Φ* degrees away from any neighboring treatment heads to meet installation space requirements. 2)The angle of the imaging system should be perpendicular to one of the treatment head angles.

To satisfy these constraints, without losing generality, we assume the angles occupied by the imaging system are set to 90° and 270°, with the first treatment head angle, *A_1_*, set to 0°. The constraints on the distribution of the remaining angles can then be expressed as Equation 4:


(4) $$A_{i}\in [0,\frac{\pi -\Phi -\Delta }{2}]\cup [\frac{\pi +\Phi +\Delta }{2},\frac{3\pi -\Phi -\Delta }{2}]\cup [\frac{3\pi +\Phi +\Delta }{2},2\pi ]$$

#### 2.3.2 Objective

The optimization objective is to minimize the discrepancy between the beam directions used by the FLASH therapy system and those in conventional clinical IMRT plans. The goal is to achieve the highest possible consistency between the beam directions generated by the FLASH system in one to three deliveries and those specified in the reference IMRT plans. In this optimization, only beam directions are considered, based on the assumption that the dose distribution achieved through dose modulation components in FLASH therapy (e.g., custom-made lead compensators) can approximate the modulated dose distribution in IMRT, which utilizes collimators and multi-leaf collimators (MLCs). Under this assumption, similar beam directions are expected to produce similar dose distributions. Although ideally, clinical objectives such as target coverage, organ-at-risk (OAR) sparing, and dose conformity should be incorporated into the optimization, doing so would significantly increase the complexity of the objective function, potentially making it intractable. Moreover, one could argue that the plan optimization process for FLASH treatments might differ in order to incorporate the FLASH effect resulted from ultra-high dose rate (19, 20), potentially resulting in different dose distributions. However, as such optimization approaches are still under investigation, this study adopts the conventional IMRT dose distribution as a reference. Within this framework, the FLASH effect occurs in regions where the dose rate is sufficiently high, thereby providing enhanced normal tissue sparing.

To illustrate how to calculate the objective function for a single IMRT plan, assume the number of beams in the plan is *M*, with corresponding beam directions *B_1_* to *B_M_*.

Case 1: M≤N . If the number of beams *M* is less than or equal to the number of treatment heads *N*, we select *M* treatment head angles $A_{1}^{*}$ to $A_{M}^{*}$ from the available *N* treatment head angles to best match the beam directions in the IMRT plan in a single delivery. There are $C_{N}^{M}$ ways to select the treatment head angles. For each selection, we determine the rotation angle *β* of the gantry to best align the selected treatment head angles with the beam directions in the treatment plan. The value of *β* is determined by minimizing the following function given in Equation 5:


(5) $$min\;f(\beta )=\sum_{i=1}^{M}\left|A_{i}^{*}+\beta -B_{i}\right|$$

where $A_{i}^{*}$ is the angle of the selected treatment head, and *B_i_* is the corresponding beam direction. The order of the treatment head angles affects the optimization result, and each possible order should be considered. The order that minimizes the objective function should be selected. There are N! possible orders to consider for each selection of treatment head angles. Equation 5 is referred to as the basic sub-optimization problem.

Case 2: *N* < *M* ≤ 2*N*. When the number of beams *M* exceeds *N* but is less than or equal to 2*N*, the *M* beams are first divided into two groups, with beam numbers *M_1_* and *M_2_*, such that *M*_1_ + *M*_2_ = *M*, and *M*_1_ ≤ *N*, *M*_2_ ≤ *N*. Two minimization problems are solved independently for the two groups of beams, with sizes *M_1_* and *M_2_*. There are $C_{M}^{M_{1}}$ ways to select the first group, leaving the remaining beams for the second group. This leads to a total of $2C_{M}^{M_{1}}$ sub-optimization problems to solve.

For example, when *N* = 5 and *M* = 6, the possible groupings for *M_1_* and *M_2_* are (1, 5), (2, 4), and (3, 3), resulting in a total of $C_{6}^{1}+C_{6}^{2}+C_{6}^{3}=36$ possible grouping schemes. For larger values of *M*, the number of possible groupings increases accordingly.

Case 3: 2*N* < *M* ≤3*N*. When *M* exceeds 2*N* but is less than or equal to 3*N*, the beams are divided into three groups, with beam numbers *M_1_*, *M_2_*, and *M_3_*, such that *M*_1_ + *M*_2_ + *M*_3_ = *M*, and *M*_1_ ≤ *N*, *M*_2_ ≤ *N*, *M*_3_ ≤ *N*. Similar to Case 2, all possible values for *M_1_*, *M_2_*, *M_3_* and their corresponding grouping schemes are considered.

Case 4: *M* > 3*N*. For *M* > 3*N*, larger groupings are unnecessary, as in practice, the beam number *M* rarely exceeds 10, which is an uncommon scenario in clinical settings. Thus, only cases where *M* ≤ 3*N* are considered.

Once the objective values for all treatment plans have been computed, the total objective function is the sum of the individual objective functions for each treatment plan, as shown in Equation 6:


(6) $$f=\sum_{j=1}^{n}\min f_{j}(\beta_{j})=\sum_{j=1}^{n}\sum_{i=1}^{M_{j}}\left|A_{i}^{*}+\beta_{j}-B_{i}\right|$$

This represents the overall discrepancy between the beam directions generated by the FLASH system and the required beam directions across all *n* treatment plans.

### 2.4 Mathematical solution to the angle selection problem and computational estimation

The optimization problem structure consists of a set of basic sub-optimization problems. For the sub-optimization problem described by Equation 5, it is easy to prove that the optimal value of *β* is given by Equation 7:


(7) $$\beta =median({\left\{A_{i}^{*}-B_{i}\right\}}_{i=1}^{n})$$

where $A_{i}^{*}$ are the treatment head angles and *B_i_* are the corresponding beam directions.

**Figure 2:**
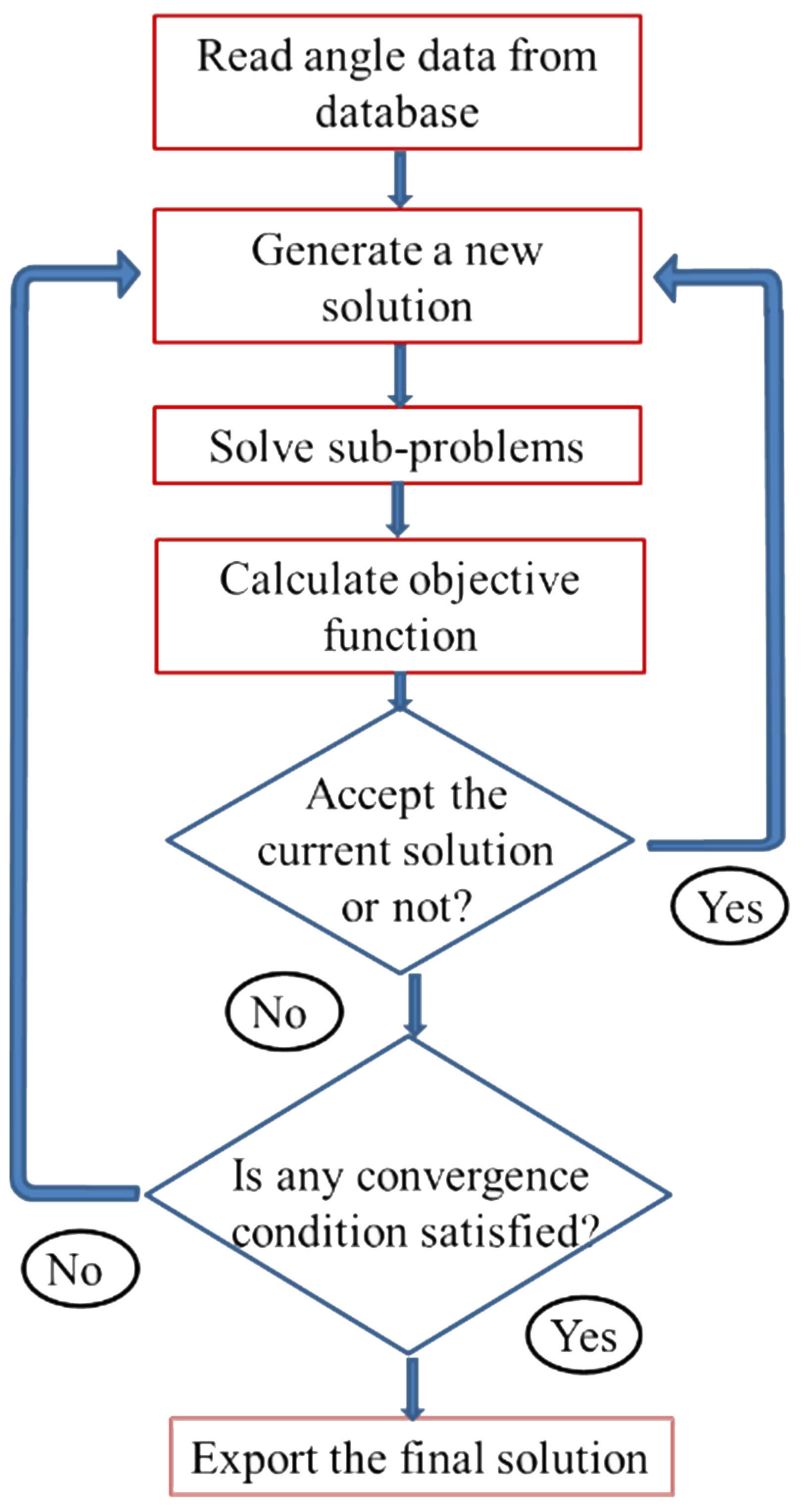
The flowchart of the overall optimization procedure for the treatment head angle arrangement optimization.

Before solving the sub-problems, one must determine the treatment head angles. These parameters are handled by the adaptive simulated annealing (ASA) algorithm (21). ASA is a global optimization algorithm employing an iteration process. In each iteration it randomly generate candidate treatment head angles using the random number generation engine of ASA. And then the enumeration method is used to list all sub-problems that are solved using Equation 7. The summation of all sub-problem objective values is the objective value of the ASA current solution. The current solution is accepted according to ASA acceptance criteria. The overall optimization procedure is shown in **Figure 2**.

During the iteration process, an enumeration method was used to list all sub-problems. Preliminary estimates suggest that the computational cost of calculating the objective function values using the enumeration method is manageable. However, more efficient computational techniques could potentially accelerate the optimization process compared to a straightforward enumeration approach.

### 2.5 Beam direction database and result estimation

To evaluate the suggested method for angle arrangement optimization, we need the patient population that will be treated using the FLASH therapy system. However these patient are difficult to predict, we used a cohort of patients treated with conventional IMRT to represent these patients. Specifically, we selected all the IMRT plans treated in our department over a two-year period before the introduction of VMAT technique. The beam directions from these treatment plans were extracted and stored in a beam direction database. This beam direction database was then used to determine the optimal treatment head angle arrangement for the FLASH radiotherapy system. The difference between the actual beam directions and the directions achieved by the system was analyzed to evaluate the performance of the angle arrangement and the optimization process.

## 3 Results

### 3.1 Details of beam direction database

The IMRT treatment plans, as optimized and delivered at our department, were obtained from our patient database. The process of establishing the beam direction database was as follows: 1) Data Collection: Beam direction data were collected from all treatment plans executed between January 2012 and January 2014. This dataset included a total of 69,928 beams from 8,866 patients. 2) Duplicate Removal: Since beams delivered by Varian machines were often split into two beams (denoted as “beam a” and “beam b”), and some plans may use beams with identical directions, any duplicate beam directions were removed (i.e., each beam direction in a plan was unique). 3) Breast Cancer Plans: For breast cancer treatment plans, which often use a special hybrid IMRT design, only the tangent beam directions were retained, and any adjacent IMRT beams to the tangent were discarded.

**Figure 3:**
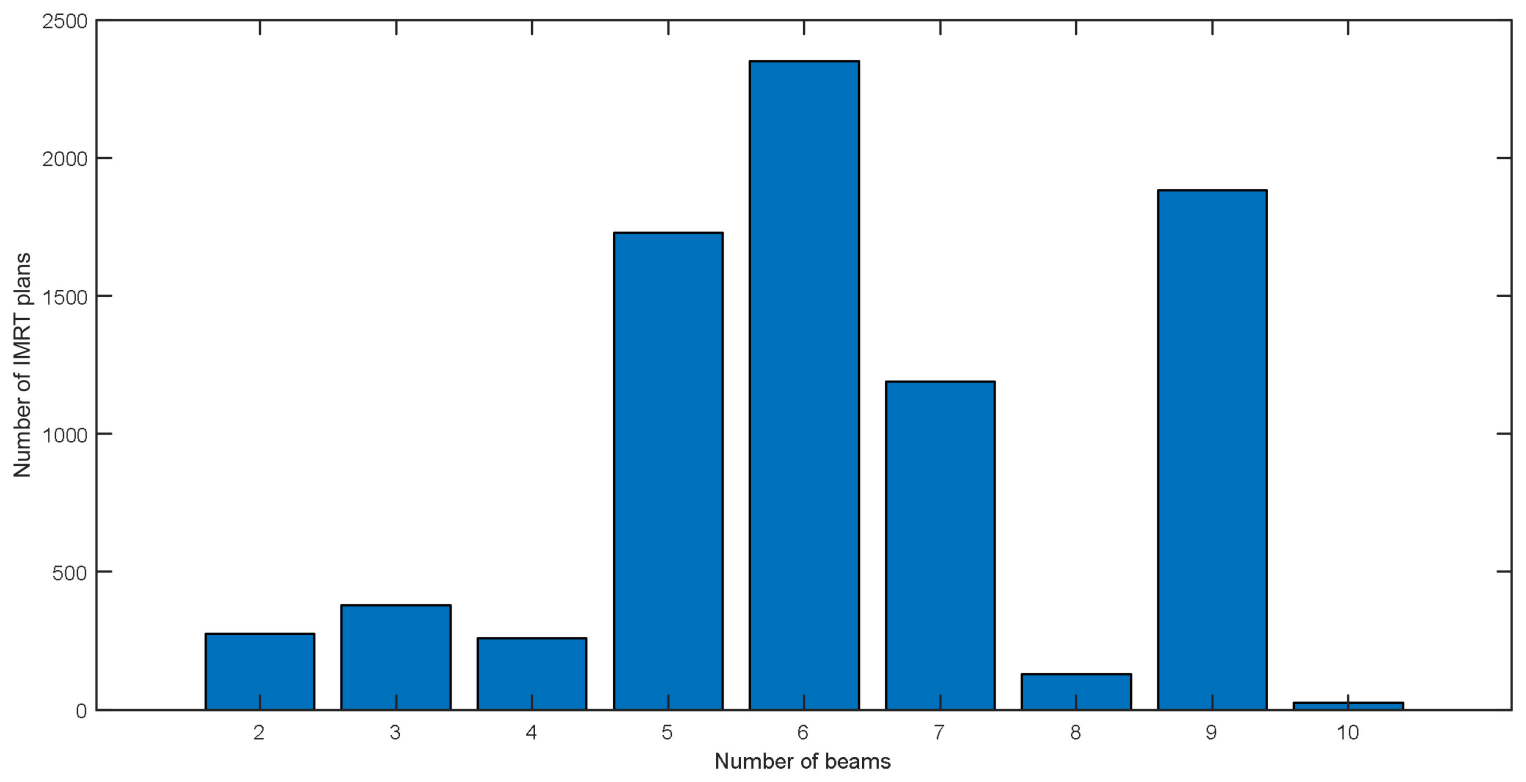
The distribution of beam numbers of all IMRT plans in the plan cohort.

After processing, the distribution of IMRT plan numbers for all used beam numbers is summarized in the **Figure 3**. The largest number of plans is 2400 for six beam plans, and the second is 1800 for nine beam plans. Among all these plans, the most frequently used beam direction arrangement is the nine equally spaced beams used in nasopharyngeal cancer treatment plans.

### 3.2 Optimized treatment head angle arrangement

For FLASH therapy platforms with five treatment heads, we obtained the optimization results both with and without the imaging system constraint shown in expression (4). When *Δ* was set to 30° and *Φ* was set to 25°, the optimized treatment head angles with the imaging system constraint, as determined by the proposed methodology, were 0°, 40.4°, 169.4°, 201.2°, and 239.8°. For this angle arrangement, the average beam direction discrepancy between the directions generated by the FLASH therapy platform and those used in the treatment plans for the plan cohort was found to be 38.9°. When *Δ* was set to 30° and *Φ* was set to 25°, the optimized treatment head angles without the imaging system constraint, as determined by the proposed methodology, were 0°, 155.4°, 234.4°, 266.2°, and 304.8°. For this optimized angle arrangement, the average beam direction discrepancy between the directions generated by the FLASH therapy platform and those used in the treatment plans for the plan cohort was found to be 37.8°.

**Figure 4:**
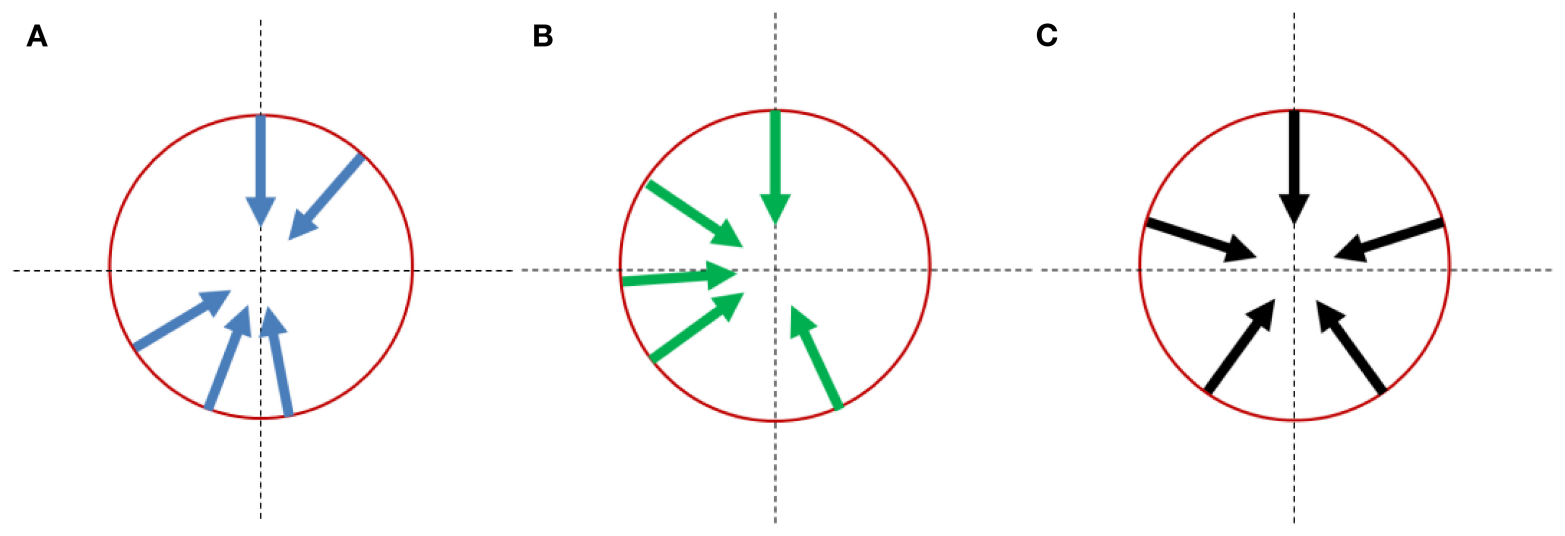
Treatment head angle arrangement for multi-angle FLASH therapy platforms: **(A)** optimizing results with the imaging system constraint; **(B)** optimizing results without the imaging system constraint. **(C)** reference arrangement with equal-spaced angles;.

As a comparison, when equally spaced angles (0°, 72°, 144°, 216°, and 288°) were used, the average beam direction discrepancy between the generated directions and the treatment plan directions for the cohort was 78.4°. The optimized treatment head angle arrangements and the equally spaced angle arrangement were shown in **Figure 4**.

### 3.3 Three simple examples

For treatment plans with five beams, the beam directions with the highest frequency were 0°, 45°, 95°, 265°, and 315°, occurring 127 times. This beam configuration is typically used in five-beam IMRT plans for rectal cancer.

For this angular distribution, the optimization program was applied directly to the five treatment head angles after an overall rotation by angle β. The value of β was calculated to be 95.6°, and the objective function value was computed to be 135.4° (**Table 1**).

**Table 1:** The beam directions for the first simple example.

Index	*1*	*2*	*3*	*4*	*5*
Beam directions in Plan (°)	265	315	0	45	95
Angles of used treatment head (°)	169.4	201.2	239.8	0	41.4
β (°)	95.6				
Cost function value (°)	135.4				

The value of β was calculated to be 95.6°, and the objective function value f was computed to be 135.4°.

**Figure 5:**
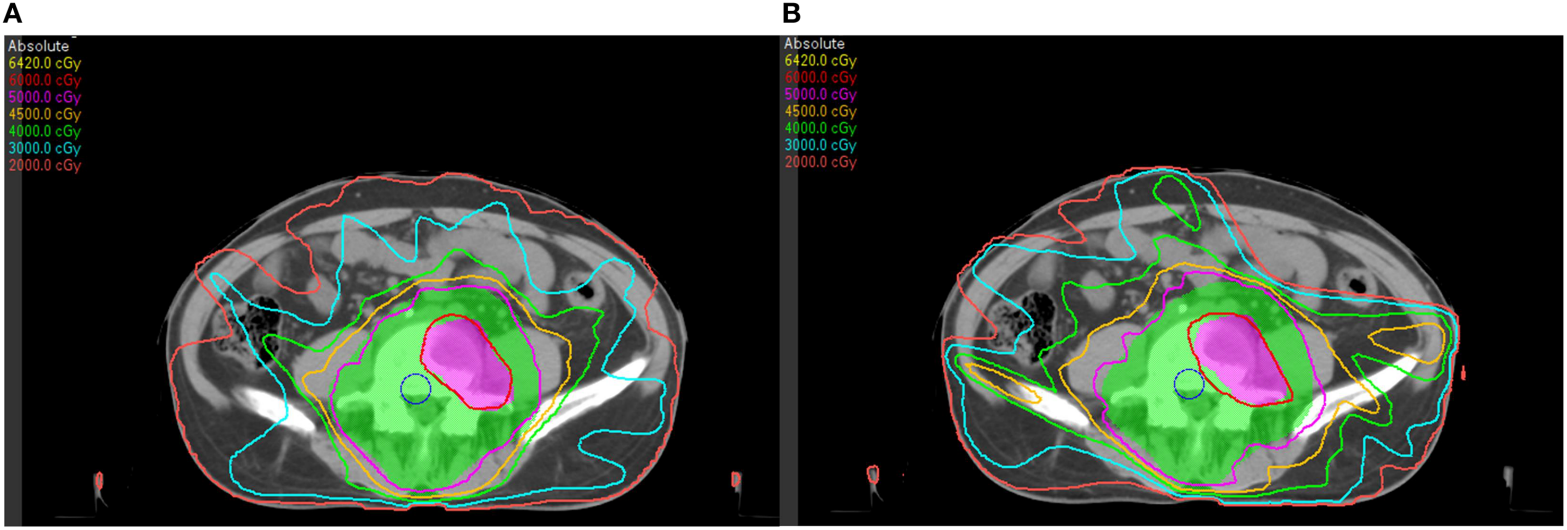
Dose distributions of a bone metastasis case: **(A)** with original beam directions; **(B)** with beam directions realized with the proposed methodology.

**Figure 6:**
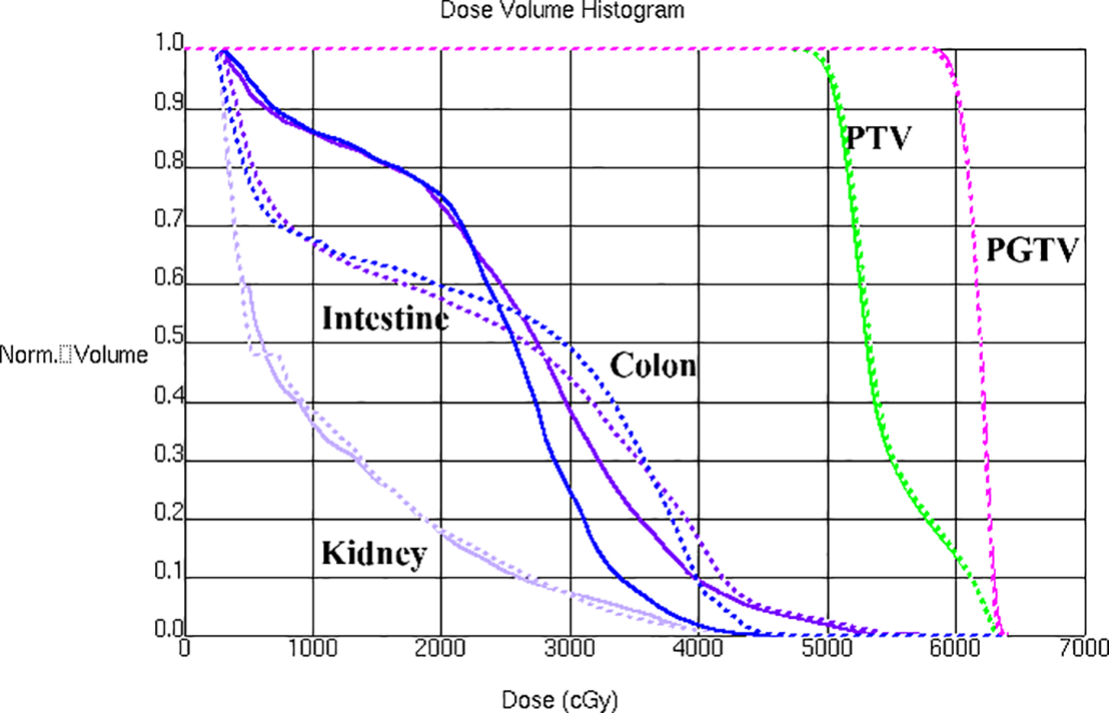
DVHs is shown for a bone metastasis case with original beam directions (solid lines) and with beam directions realized with the proposed methodology (dashed lines).

A bone metastasis case with this beam configuration was selected from the database. Dose distributions for original beam directions and the directions realized with the proposed methodology are shown in **Figure 5A, B**respectively. DVHs for this case is shown in **Figure 6**. Although the dose distributions in **Figure 5** exhibit noticeable differences, both plans demonstrate comparable target coverage. In the original plan, the maximum doses to the intestine and colon were 57.1 Gy and 44.4Gy, respectively, whereas the proposed methodology resulted in values of 55.7 Gy and 46.8 Gy for these structures. Both IMRT plans are clinically acceptable.

For treatment plans with seven beams, the most frequent beam directions were 26°, 78°, 129°, 180°, 231°, 283°, and 334°, occurring 241 times. For this distribution, the optimization program suggested a scheme involving two deliveries, using the following treatment head angles and overall rotation angle β shown in **Table 2**. The objective function value for this configuration was calculated as 34.6°.

**Table 2:** The beam directions for the second simple example.

Index		*1*	*2*	*3*	*4*
First delivery	Beam directions in Plan (°)	129	180	283	334
	Angles of used treatment head (°)	201.2	239.8	0	40.4
	β_1_ (°)	-72.2			
Second delivery	Beam directions in Plan (°)	26	78	231	
	Angles of used treatment head (°)	0	40.4	201.2	
	β_2_ (°)	29.8			
Cost function value(°)		34.6			

The value of β was calculated to be -72.2° and 29.8°, and the objective function value f was computed as 34.6°.

**Figure 7:**
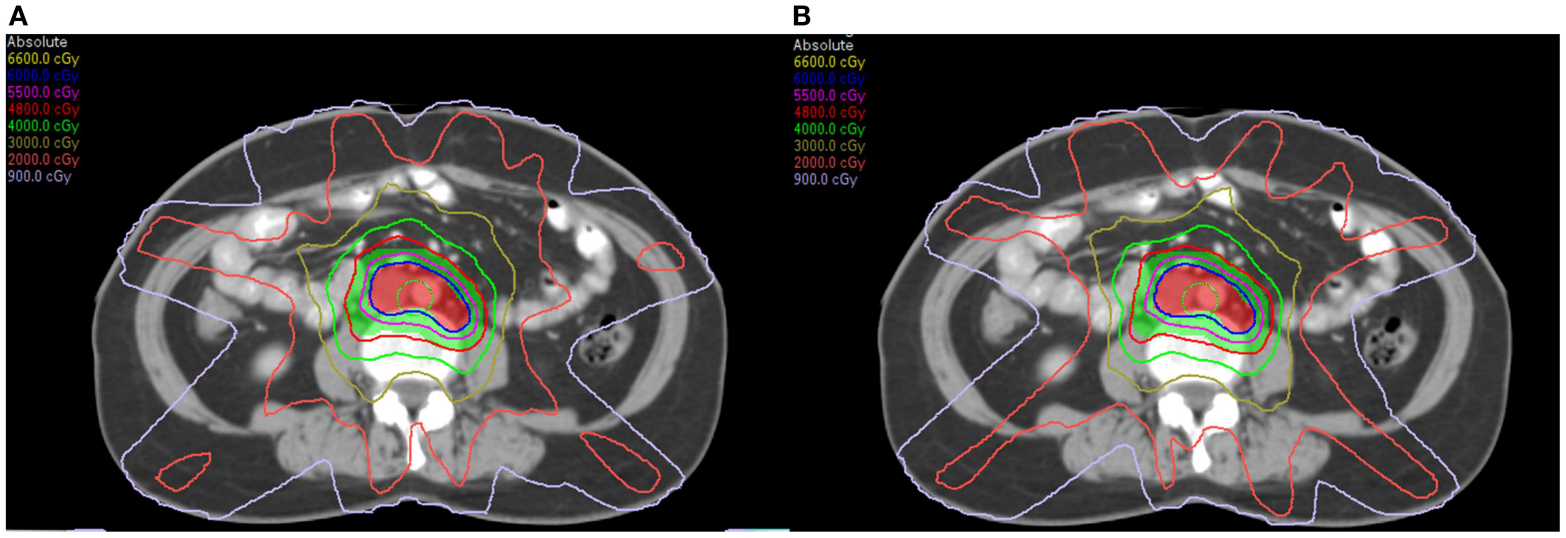
Dose distributions of a pancreas case: **(A)** with original beam directions; **(B)** with beam directions realized with the proposed methodology.

**Figure 8:**
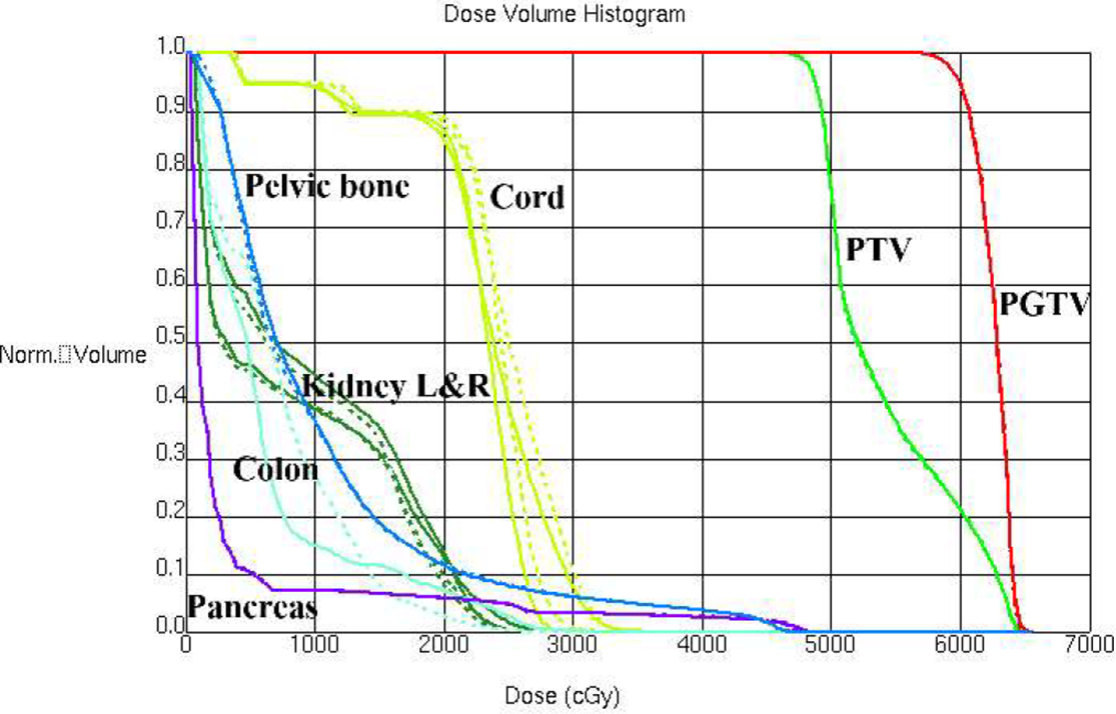
DVHs is shown for a pancreas case with original beam directions (dashed lines) and with beam directions realized with the proposed methodology (solid lines).

A pancreas case with this beam configuration was selected from the database. Dose distributions for original beam directions and the directions realized with the proposed methodology are shown in **Figures 7A, B** respectively. DVHs for this case is shown in **Figure 8**. It can be found that dose distributions and DVHs of two IMRT plans with different beam directions are nearly the same.

The final case is a nasopharyngeal case treated with a nine-beam IMRT plan. The beam directions used in this plan were 0°, 40°, 80°, 120°, 160°, 200°, 240°, 280°and 320°. For this configuration, the optimization program suggested a scheme involving two deliveries, using the following treatment head angles and overall rotation angle β shown in **Table 3**. The total angular discrepancy was 12.6°, which is estimated to have a negligible impact on plan output.

**Table 3:** The beam directions for the third simple example.

Index		*1*	*2*	*3*	*4*	*5*
First delivery	Beam directions in Plan (°)	0	40	160	200	240
	Angles of used treatment head (°)	0	40.4	169.4	201.2	239.8
	β_1_ (°)	-0.4				
Second delivery	Beam directions in Plan (°)	80	120	280	320	
	Angles of used treatment head (°)	0	40.4	201.2	239.8	
	β_2_ (°)	80				
Cost function value(°)		12.6				

The value of β was calculated to be -0.4° and 80°, and the objective function value f was computed as 12.6°.

## 4 Discussions

In this work, we proposed a methodology for determining optimal treatment head angle arrangement for multi-angle FLASH intensity modulated radiation therapy platforms that employ a limited number of treatment heads. Our results demonstrate superior performance compared to configurations with equally spaced treatment heads. It is important to note that the outcomes differ depending on whether the imaging system constraint is taken into account, and incorporating additional constraints may further alter the optimization results.

One limitation of this study is that it does not sufficiently evaluate the clinical impact of reducing angular discrepancies between the FLASH system and conventional IMRT plans. One potential approach to address this would be to conduct statistical analyses on dosimetric metrics from a cohort of treatment plans covering various tumor sites, comparing results obtained using the optimized angular configurations versus those using uniformly spaced angles. Furthermore, to quantify the biological advantage associated with the FLASH effect, regions meeting the required ultra-high dose rate threshold could be evaluated using established radiobiological models, as demonstrated in several previous studies (11, 20). These aspects will be addressed in the future work.

The global optimization algorithm ASA was employed to determine the optimal treatment head angle arrangement. In each iteration of the optimization process, an enumeration method was used to list all sub-optimization problems, which were then solved to calculate the cost function value. The complexity of this process increases rapidly with a higher number of treatment heads or beams in the treatment plans. Fortunately, because the number of treatment heads is typically fewer than six and the number of beams in IMIRT plans rarely exceeds ten, the final program running time in a personal computer equipped with Intel Core i3 of 3.2 GHz is about one hour.

A cohort of IMRT plans, collected from our clinical treatment plan database, was used to represent the plans intended for delivery using FLASH IMRT platforms. However, as FLASH research is still in progress, the tumor types suitable for FLASH treatment remain uncertain. It should be noted that the choice of plans can affect the results. Once the specific plans for FLASH IMRT are more clearly defined, they can be incorporated into the proposed methodology to determine the optimal treatment head angle arrangement.

For FLASH IMRT therapy scenarios in which the required number of beams exceeds the number of available treatment heads, it is assumed that the beams will be delivered in two or three deliveries. Further research is needed to study the effect of separate deliveries on the FLASH effect. An alternative approach to this issue is to partition the required beam directions into multiple groups and deliver them in different fractions, thereby enabling the co-optimization of the dose distribution. A detailed methodology for incorporating required beam directions to different fractions will be presented in a future publication.

One critical requirement of FLASH IMRT is that beam fluence modulation must be achieved is sub second. For the MAX-FLASH system (13, 14), this is achieved using custom-made lead compensators cast from 3D-printed molds designed according to treatment planning results. These compensators have been shown to provide improved depth dose distributions and lateral dose uniformity, improving treatment precision, as validated in dosimetric studies (22–24). However, this method is time consuming, and alternative techniques to fulfill this requirement may be necessary.

## 5 Conclusion

The methodology is capable of optimizing treatment head angle arrangement for FLASH intensity-modulated therapy platforms. This highlights the feasibility of developing intensity-modulated FLASH radiotherapy plans that can be efficiently delivered using a multi-angle photon FLASH system.

## Data Availability

The raw data supporting the conclusions of this article will be made available by the authors, without undue reservation.
